# Rapid Identification of Intact Staphylococcal Bacteriophages Using Matrix-Assisted Laser Desorption Ionization-Time-of-Flight Mass Spectrometry

**DOI:** 10.3390/v10040176

**Published:** 2018-04-04

**Authors:** Dana Štveráková, Ondrej Šedo, Martin Benešík, Zbyněk Zdráhal, Jiří Doškař, Roman Pantůček

**Affiliations:** 1 Department of Experimental Biology, Faculty of Science, Masaryk University, Kotlářská 2, 61137 Brno, Czech Republic; dana.stverak@mail.muni.cz (D.Š.); martin.benesik@mail.muni.cz (M.B.); doskar@sci.muni.cz (J.D.); 2 Central European Institute of Technology, Masaryk University, Kamenice 5, 62500 Brno, Czech Republic; sedo@post.cz (O.Š.); zbynek.zdrahal@ceitec.muni.cz or zdrahal@sci.muni.cz (Z.Z.); 3 National Centre for Biomolecular Research, Faculty of Science, Masaryk University, Kamenice 5, 62500 Brno, Czech Republic

**Keywords:** MALDI-MS, *Staphylococcus*, bacteriophages, phage therapy, *Kayvirus*, Viral proteins

## Abstract

*Staphylococcus aureus* is a major causative agent of infections associated with hospital environments, where antibiotic-resistant strains have emerged as a significant threat. Phage therapy could offer a safe and effective alternative to antibiotics. Phage preparations should comply with quality and safety requirements; therefore, it is important to develop efficient production control technologies. This study was conducted to develop and evaluate a rapid and reliable method for identifying staphylococcal bacteriophages, based on detecting their specific proteins using matrix-assisted laser desorption ionization time-of-flight mass spectrometry (MALDI-TOF MS) profiling that is among the suggested methods for meeting the regulations of pharmaceutical authorities. Five different phage purification techniques were tested in combination with two MALDI-TOF MS matrices. Phages, either purified by CsCl density gradient centrifugation or as resuspended phage pellets, yielded mass spectra with the highest information value if ferulic acid was used as the MALDI matrix. Phage tail and capsid proteins yielded the strongest signals whereas the culture conditions had no effect on mass spectral quality. Thirty-seven phages from *Myoviridae*, *Siphoviridae* or *Podoviridae* families were analysed, including 23 siphophages belonging to the International Typing Set for human strains of *S. aureus*, as well as phages in preparations produced by Microgen, Bohemia Pharmaceuticals and MB Pharma. The data obtained demonstrate that MALDI-TOF MS can be used to effectively distinguish between *Staphylococcus*-specific bacteriophages.

## 1. Introduction

Due to increasing antibiotic resistance, bacterial infections have become a serious problem in hospital and community environments. A possible approach to combat such infections is phage therapy, either as an alternative to antibiotics or utilizing phage-antibiotic synergy. Experimental phage therapy proved to be successful for the treatment of bacterial infections in animal models and human patients [1]. Interest in phage therapy has therefore increased [2] and applications of phages are currently being investigated extensively [3]. For the rational use of phages, it is necessary to have rapid methods of identification, particularly for new isolates as well as previously characterized phages after passaging for quality control of bacteriophage-based products.

Identification of bacteriophages is usually based on morphological characterization using electron microscopy and by genome sequencing [4]. Multiplex PCR can be used to identify well-characterized phages with defined conserved genes typical for particular groups, for example, phages of dairy bacteria [5] or staphylococcal phages [6]. However, high mosaicism and modular genomic structure of tailed phages complicates their detection [7].

This work focuses on the identification of staphylococcal phages that play an important role in biology, evolution and pathogenicity of staphylococci [8,9,10,11,12]. While lytic phages shape bacterial population dynamics, temperate phages are a driving force in bacterial evolution and can benefit the host bacteria by introducing novel traits such as virulence factors as a consequence of lysogenic conversion [13]. Bacteriophages also facilitate the horizontal transfer of bacterial DNA, including resistance genes, through transduction [14,15,16].

The taxonomy of staphylococcal phages has recently been updated by the International Committee on Taxonomy of Viruses (ICTV) [17]. All staphylococcal phages belong to the order *Caudovirales,* including *Myoviridae*, *Siphoviridae* and *Podoviridae* families. Staphylococcal phages from the family *Siphoviridae* are temperate and comprise 3 genera that correlate with previously described serological groups [18]. Candidates for combating staphylococcal infections are phages belonging to *Myoviridae* and *Podoviridae* families [8] as they seem unable to lysogenise host cells [11] and recent findings suggest that lytic and polyvalent staphylococcal phages belonging to the *Myoviridae* family are safe for therapeutic applications [19].

Fingerprinting by means of Matrix-Assisted Laser Desorption/Ionization—Time-of-Flight Mass Spectrometry (MALDI-TOF MS) is based on rapid detection of compounds ionized directly or after minimal sample treatment. The greatest success of this method has been achieved in the field of bacterial identification [20]. Protein profiles obtained by MALDI-TOF MS are used nowadays in thousands of clinical laboratories worldwide. Apart from bacteria, several other types of samples, including viruses, can be subjected to MALDI-TOF MS profiling analysis [21]. However, MALDI-TOF MS profiling is not currently used for routine identification of tailed phages.

The first successful attempt at bacteriophage MALDI-TOF MS profiling was documented by Thomas et al. [22]. This study focused on the ssRNA phage MS2 that belongs to the *Leviviridae* family and infects *Escherichia coli*. Treatment of MS2 phage particles, isolated by centrifugation and ultrafiltration, with 50% acetic acid was found to be necessary to obtain protein signals, as an acidic environment causes disassembly of phage protein structures. Acid treatment directly in the MALDI matrix solution was also found to be beneficial, as demonstrated by the successful generation of protein signals after preparing the phage sample in a MALDI matrix comprising 17% formic acid [23,24]. To improve reproducibility and sensitivity, McAlpin et al. [25] suggested a 10 min treatment of *Yersinia pestis* podovirus φA1122 with β-mercaptoethanol to reduce inter-molecular disulphide bonds. This procedure was found to be more efficient than acid treatment.

Despite relatively long-standing knowledge that MALDI-TOF MS fingerprinting of bacteriophages was possible, most studies in the field were aimed at method optimization and involved only a limited set of strains. The largest collection of 12 strains was assessed by Bourdin et al. [26] who used MALDI-TOF MS profiling for phage purification control after a multi-step isolation procedure. These works have demonstrated the usefulness of phage protein profiling for particular analytical purposes, however, comprehensive evaluation of sample preparation methods, MALDI-TOF mass spectral quality, reproducibility and discriminatory power have not yet been assessed critically. For this reason, we conducted a study on a relatively large set involving closely related staphylococcal phage strains, using various combinations of sample preparation techniques to establish the optimum setup and assess its applicability in identifying bacteriophages.

## 2. Materials and Methods

### 2.1. Bacterial Strains and Phages

Phages and bacterial strains used in this work are described in Table 1. The bacteriophages from the International Typing Set for human *S. aureus* and their propagation strains (PS) were obtained from Dr. P. Petráš (National Reference Laboratory for *Staphylococci*, National Institute of Public Health, Prague, Czech Republic). Laboratory lysogenised strains PS 47[53^+^], PS 47[77^+^] were prepared previously [27]. Phages 11, 80α and K and *S. aureus* RN4220 were kindly provided by Prof. C. Wolz (Interfaculty Inst. of Microbiology and Infection Medicine Tübingen, University of Tübingen, Tübingen, Germany). Propagation strain *S. aureus* RN4220 *ΔtarM* for podoviruses [28] was provided by Prof. A. Peschel (Department of Infection Biology, University of Tübingen, Tübingen, Germany). Phages Twort, 44AHJD and P68 were purchased from Félix d’Hérelle Reference Center for Bacterial Viruses (Université Laval, Québec, QC, Canada) and phage X2 from the National Collection of Type Cultures (Public Health England, Salisbury, UK). Phages B166 and B236 [29], 131 and 812 [30] and phage K1/420 [31] were described previously. Phage PYO was isolated from a phage cocktail containing *Staphylococcus*, *Streptococcus*, *Proteus*, *E. coli* and *Pseudomonas aeruginosa* phages (PYO Bacteriophagum combinierae liquidum, lot no. 000860, BioPharm, Tbilisi, Republic of Georgia). A commercial *Staphylococcus* bacteriophage preparation produced by Microgen (lot no. H141, 0812, 08.14, Moscow, Russia) was purchased in a pharmacy in Samara, Russia. Bacteriophage preparation Stafal^®^ lot no. 140805201 was kindly provided by Bohemia Pharmaceuticals (Prague, Czech Republic). Phage preparation Duofag, staphylococcal phages SAU1 and SAU2 and *P. aeruginosa* phage PAE1 were kindly provided by MB Pharma (Prague, Czech Republic). *S. aureus* strains CCM 4028, CCM 4890 and CCM 8428 were obtained from the Czech Collection of Microorganisms (Masaryk University, Brno, Czech Republic).

### 2.2. Bacteriophage Propagation and Titration

Phages were propagated on their propagation strains (Table 1) in a liquid medium meat-peptone broth (MPB) prepared from 13 g of nutrient broth (Oxoid, CM0001), 3 g of yeast extract (Oxoid, LP0021) and 5 g of peptone (Oxoid, LP0037) dissolved in distilled water to 1000 mL (pH 7.4). Prophage-less *S. aureus* strains CCM 4890 [32], RN4220 [33] and CCM 8428 [34] were used for propagation of phages with known genomic sequences to eliminate the possibility of false positive signals in MALDI-TOF MS spectra caused by induced prophages. Phages 29, 42E and 79 were propagated in a soft agar layer with 0.7% (*w*/*v*) of bacteriological agar (Oxoid, LP0011) on top of solid meat-peptone agar with 1.5% (*w*/*v*) technical agar (Oxoid, LP0012). Two other types of growth media were used to test the influence of medium on mass spectral quality: brain heart infusion (BHI) broth (Oxoid, CM1135; pH 7.4) and 2× yeast-tryptone broth (2YT) consisting of 16 g of tryptone (Oxoid, LP0042), 10 g of yeast extract (Oxoid, LP0021) and 5 g of NaCl dissolved in distilled water to 1000 mL (pH 7.4).

For phage titration, ten-fold serial dilutions of phage in MPB were prepared. An overnight culture of propagation strain in MPB was mixed with 1/10 volume of 0.02 M CaCl_2_. Bacterial suspension (0.1 mL) was added to 3 mL of 0.7% meat-peptone soft agar cooled to 45 °C and overlaid on 1.5% meat peptone agar plates. The plates were left to dry for 10 min. Phage dilutions were dropped onto the top agar and left to dry. Plates were incubated overnight at 37 °C.

### 2.3. Concentrating Bacteriophage to Pellets

Phage lysate, obtained after complete lysis of the bacteria, was centrifuged at 4500× *g* for 30 min at 4 °C and filtered through 0.45 μm pore-sized polyethersulfone syringe filters (Techno Plastic Products, Trasadingen, Switzerland) to remove bacterial debris. Phages were pelleted by centrifugation at 54,000× *g* for 2.5 h at 4 °C in a JA-30.50 Ti rotor (Beckman, Brea, CA, USA). For preparation of pellets from 3 mL of phage lysates, conical tubes (part no. 358119, Beckman) and adapters (part no. 358153, Beckman) with an SW 55 Ti rotor (Beckman) were used. The resulting pellet was resuspended in 350 μL of phage buffer (5 × 10^−2^ mol/L Tris pH 8.0, 10^−2^ mol/L CaCl_2_, 10^−2^ mol/L NaCl) overnight at 4 °C. For resuspending the pellets from 3 mL samples, 30 μL of phage buffer was used.

### 2.4. Purification of Phage Particles by CsCl Density Gradient Centrifugation

This procedure was carried out according to the description by Nováček et al. [31]. Phage pellets resuspended in phage buffer were used as an input material for CsCl density gradient centrifugation. Soluble proteins from resuspended pellets were removed by extraction with an equal volume of chloroform. The resulting aqueous fraction (approximately 1.2 mL) was overlaid onto a preformed CsCl (Sigma-Aldrich, St. Louis, MO, USA) density gradient (1 mL of each 1.45 g/mL 1.50 g/mL, 1.70 g/mL of CsCl in phage buffer) and centrifuged at 194,000× *g* for 4 h at 12 °C using a SW 55 Ti rotor (Beckman). Phage particles forming a visible zone were collected by puncturing the tube with an 0.8 mm gauge needle and syringe. Caesium chloride was removed from the phage-containing fraction by dialysis against an excess of phage buffer at 4 °C overnight using Visking dialysis tubing type 8/32”, 0.05 mm thick (part no. 1780.1, Carl Roth, Karlsruhe, Germany).

### 2.5. Fast Protein Liquid Chromatography (FPLC)

Bacteriophage lysates were purified using a monolithic column, CIMmultus^TM^ QA 1 mL (Bia separations, Ajdovščina, Slovenia) and FPLC NGC (Bio-Rad, Hercules, CA, USA). Phages were purified according to column manufacturer’s recommendation with minor modifications [35]. After removing bacterial debris by centrifugation and filtration, 25 mL of phage lysate (10^8–9^ PFU/mL) were mixed with 100 mM sodium phosphate buffer pH 7 at a ratio of 1:1. The column was equilibrated with 100 mM sodium phosphate buffer pH 7. The phage suspension was loaded onto the column and a linear gradient of 100 mM sodium phosphate, 2 M NaCl, pH 7 buffer was applied for elution of phages. NaCl was removed from the phage-containing fraction by dialysis as described following CsCl density gradient centrifugation.

### 2.6. Phage Purification by Ultrafiltration

Filtered phage lysate was used for tangential flow filtration using Pellicon XL 50 Ultrafiltration Cassettes (Millipore, Burlington, MA, USA) Biomax 300 (for *Podoviridae* and *Myoviridae* phages) and Biomax 500 (for *Siphoviridae* phages) according to the manufacturer’s instructions. All fluids were loaded onto the cassette with a peristaltic pump set at 40–50 mL per minute. The cassette was rinsed with 300 mL of sterile distilled water and then 300 mL of phage buffer. 350 mL of phage lysate was loaded onto the cassette and after concentrating/thickening of phages to 10–50 mL, phages were mixed with 1000 mL of phage buffer and thickened again to 10–50 mL.

### 2.7. Phage Precipitation by Polyethylene Glycol (PEG)

Phages were purified with PEG 8000 (Sigma-Aldrich) according to Sambrook et al. [36] with minor modifications. NaCl and PEG 8000 were dissolved in 30 mL of filtered phage lysate to final concentrations of 0.5 M and 10 % (*w*/*v*), respectively, by brief stirring. Phages were precipitated overnight at 4 °C, pelleted by centrifugation at 10,000× *g* for 15 min at 4 °C and the supernatant was discarded. Pellets were dissolved in 0.5 mL of phage buffer overnight at 4 °C. Phage particles were then separated from co-precipitated bacterial debris by centrifugation at 5000× *g* for 10 min at 4 °C. The residual PEG was removed by gentle extraction for 1 min with an equal volume of chloroform. The phage-containing aqueous phase was separated by centrifugation at 5000× *g* for 15 min, collected and filtered through 0.22 μm pore-sized polyethersulfone syringe filters (Techno Plastic Products, Trasadingen, Switzerland).

### 2.8. Sample Preparation for MALDI-MS

The phage samples were mixed with a MALDI matrix solution in a 1:4 *v*/*v* ratio, the resulting mixtures were applied to three positions on the stainless steel MALDI sample plate in a volume of 0.6 μL and were allowed to dry at room temperature. Ferulic acid (FerA, 12.5 mg/mL in a water:acetonitrile:formic acid, 50:33:17, *v*/*v* mixture), or, alternatively, alpha-cyano-4-hydroxycinnamic acid (HCCA, saturated solution in water:acetonitrile:trifluoroacetic acid, 47.5:50:2.5, *v*/*v* mixture) were used as the MALDI matrix solutions.

For peptide mass fingerprinting and MS/MS analyses, the phage preparations in a volume of 5 μL were incubated with 50 ng of trypsin in 25 mM ammonium bicarbonate for two hours at 40 °C. The proteolytic mixture in a volume of 1 μL was applied to an AnchorChip MALDI sample plate, mixed with 0.6 μL of the MALDI matrix (alpha-cyano-4-hydroxycinnamic acid, 2 mg/mL in a water:acetonitrile:trifluoroacetic acid, 32:66:2, *v*/*v* mixture) and allowed to dry at room temperature.

### 2.9. MALDI-MS Profiling Analysis

MALDI-TOF mass spectral fingerprints were obtained using an Ultraflextreme instrument (Bruker Daltonik, Bremen, Germany) operated in the linear positive mode under FlexControl 3.4 software. External calibration of the mass spectra in the linear positive mode was performed using lysozyme (its monomer, dimer and multiply protonated ions). Laser power was set to 120% of the threshold laser power for a particular type of sample. Three independent spectra comprising 1000 laser shots each were acquired from each of the wells. Within an individual well, a minimum of 200 and a maximum of 400 shots were obtained from one position. The mass spectra were recorded within the *m/z* range of 2–100 kDa. Mass spectra were processed using Flex Analysis (version 3.4; Bruker Daltonik). The MALDI-TOF mass spectra-based dendrogram was constructed using the Pearson’s product moment coefficient as a measure of similarity and the unweighted pair group average linked method (UPGMA) as a grouping method using the Biotyper 3.1 software (Bruker Daltonik). The protein fingerprints of phages with known genomic sequences were closely examined by comparing the *m/z* values with the predicted molecular weight (Mw) of phage structural proteins from the NCBI Protein database or from custom RAST annotations [37]. For matching the *m/z* values to Mw of phage structural proteins, a 500 ppm tolerance was taken into account. The first amino acid of a protein was identified using TermiNator [38,39] and the Mw was calculated using ExPASy ProtParam [40].

### 2.10. MALDI-MS/MS Analysis

Analyses of digested samples were carried out using the same instrument operated in the reflectron positive mode. Seven peptide standards (Bruker Daltonik) covering the mass range of 700–3100 Da were used for external mass calibration. Peptide maps were acquired with 800 laser shots. Peaks with minimum S/N = 10 were picked out for MS/MS analysis employing the LIFT arrangement with 600 laser shots for each peptide. The MASCOT 2.2 (MatrixScience, London, UK) search engine was used for processing the MS and MS/MS data. Database searches were carried out on the NCBIprot database (non-redundant, taxonomy All Entries; downloaded from ftp://ftp.ncbi.nih.gov/blast/db/FASTA/). For MALDI-MS data, a mass tolerance of 30 ppm was allowed for peptide mapping and 0.5 Da for MS/MS ion searches. Oxidation of methionine as an optional modification and two enzyme miscleavages were set for all searches. Peptides with statistically significant peptide scores (*p* < 0.05) were considered. Manual MS/MS spectral assignment validation was carried out.

## 3. Results

### 3.1. Comparison of Phage Purification Techniques for Sample Preparation and MALDI-TOF MS Method Development

**Figure 1:**
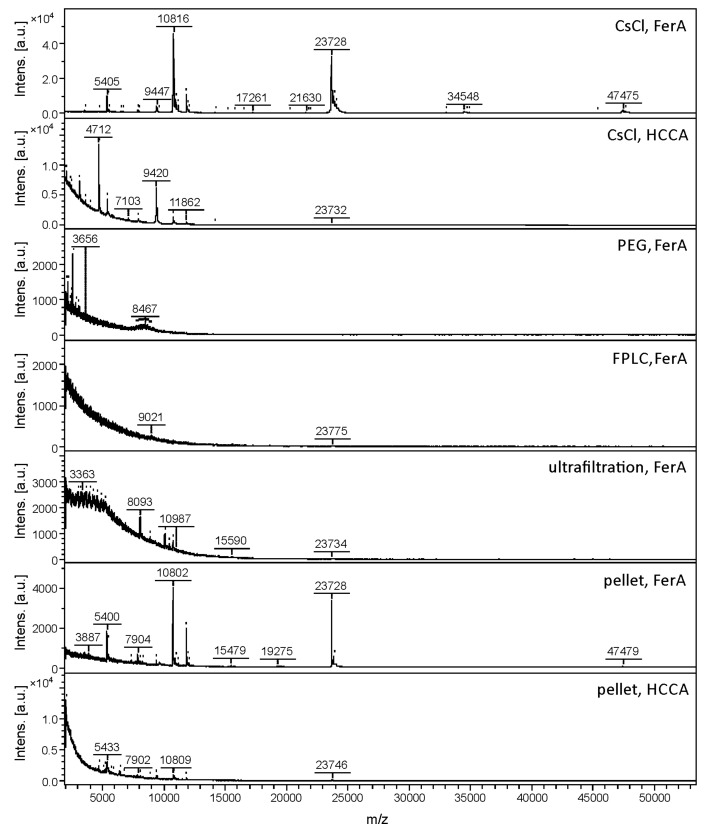
Comparison of the mass spectra of phage 77 prepared by five different sample preparation methods and by using two different matrix-assisted laser desorption ionization matrices, ferulic acid (FerA) and alpha-cyano-4-hydroxycinnamic acid (HCCA) shown for samples prepared by CsCl density gradient centrifugation and pellet dissolution.

Five different techniques that are commonly used for phage purification and concentration were tested together with two MALDI matrices for developing a rapid and reliable MALDI-TOF MS-based method for identification of *S. aureus* bacteriophages. Traditional techniques used for the concentration and purification of phages involve centrifugation, filtration, precipitation and zonal ultracentrifugation [41]. In addition to these traditional techniques, FPLC has been recently proved to be efficient for phage purification [42]. The following types of phage samples were analysed by MALDI-TOF MS: (i) phage pellets dissolved in phage buffer; (ii) phages purified by CsCl density gradient centrifugation; (iii) PEG precipitated phages; (iv) phages purified using FPLC and a poly(glycidyl methacrylate-co-ethylene dimethacrylate) monolith column modified with quaternary amine groups; and (v) phages concentrated and purified with Pellicon XL 50 ultrafiltration cassettes. As a measure of the feasibility of the sample preparation method, the mass spectral quality, in terms of the number of peaks detected, the signal-to-noise ratio and reproducibility based on three independent experiments separated by a minimum of weekly intervals, were assessed on five phage specimens belonging to five different genera from three families: phage K1/420 belonging to the *Myoviridae* family and *Kayvirus* genus; phage P68 belonging to the *Podoviridae* family and the *P68virus* genus; phages 3A, 71 and 77 belonging to the *Siphoviridae* family and *Triavirus*, *Phietavirus* and *Biseptimavirus* genera, respectively. The results are summarized in Table 2 and depicted in Figure 1.

Caesium chloride density gradient centrifugation provided reproducible MALDI-TOF mass spectra with the highest number of peaks and signal-to-noise ratios for most of the five samples assessed. This method is the most precise for phage purification and is routinely used in structural studies of phages. On the other hand, CsCl density gradient centrifugation is an expensive and time-consuming method that is inaccessible to many users and inapplicable for large scale production of phages. Therefore, MALDI-TOF MS phage identification using other affordable phage purification methods was investigated. PEG precipitation is an easy method for small-scale phage purification but the residues of PEG 8000 in samples interfered with the ionization of proteins, so this method was determined to be unsuitable for subsequent MALDI-TOF MS profiling analysis (Figure 1). When FPLC was used for phage purification, the mass spectra showed approximately 50% of signals compared to spectra of the CsCl purified phages. Similarly, ultrafiltration was not determined to be a suitable method, yielding a considerably lower number of signals (Table 2). To improve the mass spectral quality, treatment of the phages with β-mercaptoethanol for disassembling the phage particles was assessed, as described by McAlpin et al. [25]. Apart from the recommended protocol (10 min treatment at a 1:10 *v*/*v* ratio), a treatment of phage 3A purified by 500 kDa Pellicon XL 50 ultrafiltration cassette was modified to 30 or 60 min at *v*/*v* ratio of 1:5 or 1:30. However, no improvement in mass spectral quality was achieved by any of the β-mercaptoethanol treatment conditions tested (Figure S1).

The simplest setup consisting of phage pellet dissolution in phage buffer yielded surprisingly high-quality spectra comparable to those acquired from phages purified by CsCl density gradient centrifugation. Although in this case, the phage was not completely separated from bacterial cell debris, the quantity of phage particles was sufficient to provide mass spectra containing the majority of peaks (80–90%) corresponding to those detected after CsCl purification (Figure 1). Other components of the pellet, such as remaining bacterial cell debris evidently do not cause interference by suppressing ionization of the bacteriophage proteins nor by the appearance of superfluous signals unrelated to bacteriophages.

**Figure 2:**
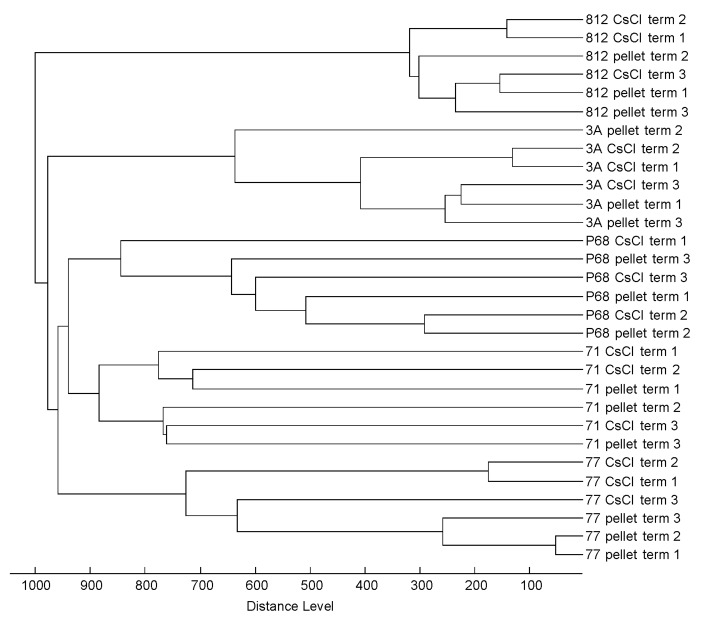
Cluster analysis based on matrix-assisted laser desorption ionization-time-of-flight (MALDI-TOF) mass spectra of three independent analyses of five phage strains prepared by CsCl density gradient centrifugation and pellet dissolution.

The influence of sample preparation technique on mass spectral quality was assessed in more depth by cluster analysis involving mass spectra obtained by three repeated analyses, using both CsCl density gradient centrifugation and pellet dissolution (Figure 2). The distances corresponding to variability among three repeated experiments were greater than those arising from different sample preparation methods. The influence of sample preparation method on analytical output was found to be insignificant. The simpler sample preparation procedure thus yielded satisfactory mass spectral outputs; however, to accurately determine the relationship between mass spectra and phage protein components, CsCl density gradient centrifugation was the most appropriate.

A disadvantage of the MALDI-MS profiling method using ferulic acid as the MALDI matrix was the inability to achieve automatic mass spectral acquisition due to non-homogeneous crystallization on the MALDI target. For that reason, another matrix solution (HCCA) was tested, selected on the basis of results of Bourdin et al. [26]. This matrix solution is also used for routine bacterial identification; its use in phage profiling would thus be more compatible with a microbiological laboratory workflow. The mass spectra obtained using HCCA as a matrix revealed signals in a narrower mass range (Figure 1). In addition, in some cases, almost all peaks detected corresponded to different ionization forms of the same protein caused by multiple protonation or gas-phase oligomer formation. The information content of the mass spectra was therefore limited, negatively influencing the discriminatory power of the method. In addition, the use of HCCA as a matrix was not advisable in combination with samples prepared by pellet dissolution, as all mass spectral quality parameters were significantly deteriorated in that case (see Figure 1 and Table 2). For that reason, ferulic acid as a MALDI matrix, in combination with phage pellet dissolution, was used in the remaining profiling experiments.

### 3.2. Effect of Culture Conditions on the Quality of Phage MALDI-TOF Mass Spectra

**Figure 3:**
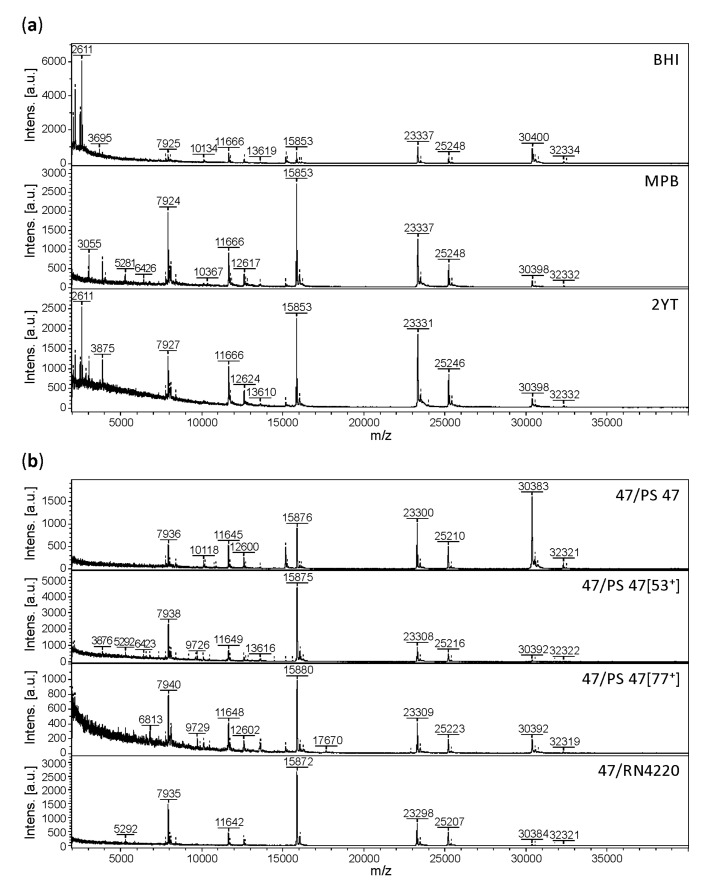
Comparison of the mass spectra of phage 3A using three different cultivation media (**a**) and phage 47 grown on four different propagation strains (**b**).

**Figure 4:**
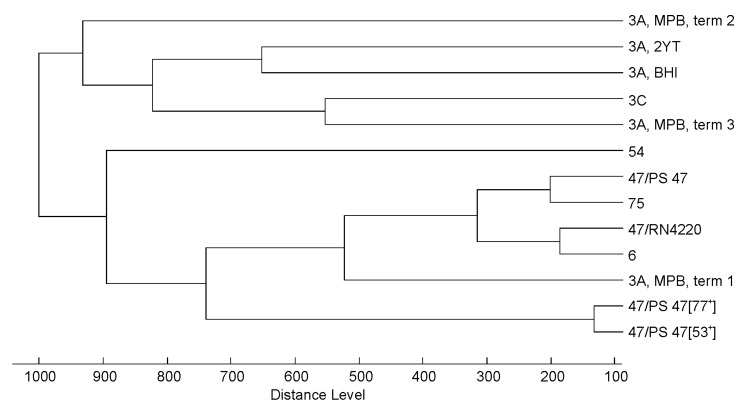
Cluster analysis of *Triavirus* mass spectra demonstrating the influence of the mass spectral variability of the method itself (phage 3A, term 1, term 2, term 3), variability resulting from different propagation strains (phage 47 propagated on *S. aureus* RN4220, PS 47, PS 47 [53^+^] and PS 47 [77^+^]) and different cultivation media (phage 3A propagated in meat-peptone broth (MPB), brain heart infusion (BHI) and 2× yeast-tryptone broth (2YT).

Culture conditions can affect bacterial MALDI-MS protein fingerprints. As published previously [43], there were small differences in the MALDI-TOF mass spectra of bacteria cultured under different conditions. Nevertheless, despite those differences, whole bacterial cells were identifiable by MALDI-TOF MS regardless of the medium used. However, it is not known whether phage proteomes are influenced by different phage propagation conditions. As a parameter of sample preparation, the influence of culture medium on MALDI-TOF mass spectral quality was tested. For that purpose, the five phage strains 3A, 71, 77, K1/420 and P68 used for method development were grown on prophage-less *S. aureus* strains in three different growth media (MPB, BHI and 2YT). As an example, the mass spectra of phage 3A are compared in Figure 3a. The main differences between the mass spectra were the relative intensities of signals in the high-mass range. Differences were also visible in the low-mass range (especially at *m/z* lower than 3.5 kDa). These probably resulted in more distant positions of the analyses in the dendrogram (Figure 4), equivalent to mass spectral differences due to the accuracy of the method itself (distance level equal to 1000). Similar effect of the culture conditions on the mass spectral quality was determined also for the remaining four strains 71, 77, K1/420 and P68 showing only minor qualitative differences in their mass spectra. The proportion of peaks conserved regardless of the cultivation conditions was greater than 80% in all cases including nine replicate analyses per sample.

The influence of different *S. aureus* strains used for phage propagation was also tested, especially for the possibility of induced prophages that could affect the protein fingerprint obtained by MALDI-TOF MS. Bacteriophage 47 was grown on prophage-less strain RN4220, on strain PS 47 (=RN1) known to carry three prophages 11, 12 and 13 and on two quadruple lysogenic strains derived from PS 47 harbouring additional prophages 53 or 77. The MALDI-TOF mass spectra shown in Figure 3b reveal that the vast majority of peaks were conserved, regardless of the propagation strain. Qualitative mass spectral differences were associated with peaks of low intensity in the low-mass range (lower than 7 kDa), representing less than 15% of all peaks observed in the individual mass spectra. The most significant differences were of a quantitative nature, where the relative intensities of peaks at *m/z* greater than 20 kDa and their signal-to-noise ratios, were influenced by the propagation strain.

The impact of propagation strain on the discriminatory power of the method was tested by means of cluster analysis based on MALDI-TOF mass spectra of six *Triavirus* strains of the *Siphoviridae* family that have very similar MALDI-TOF mass spectra (Figure 4). While the accuracy of the method, as reflected by the distance between three independent analyses of phage strain 3A, reached the maximum dendrogram distance (1000), mass spectral differences of phage 47 arising from different propagation strains resulted in a distance of 750. Note that due to the clustering method used (see Section 2.9), these threshold values are dependent of the collection of mass spectra used for the generation of individual dendrograms and the distance level representing the minimal similarity is always considered to be the highest distance (1000). The influence of propagation strain was thus lower than the overall accuracy of the method and its impact on the discriminatory power of the method was practically insignificant.

### 3.3. Profiling of 37 Phages by MALDI-TOF MS

**Figure 5:**
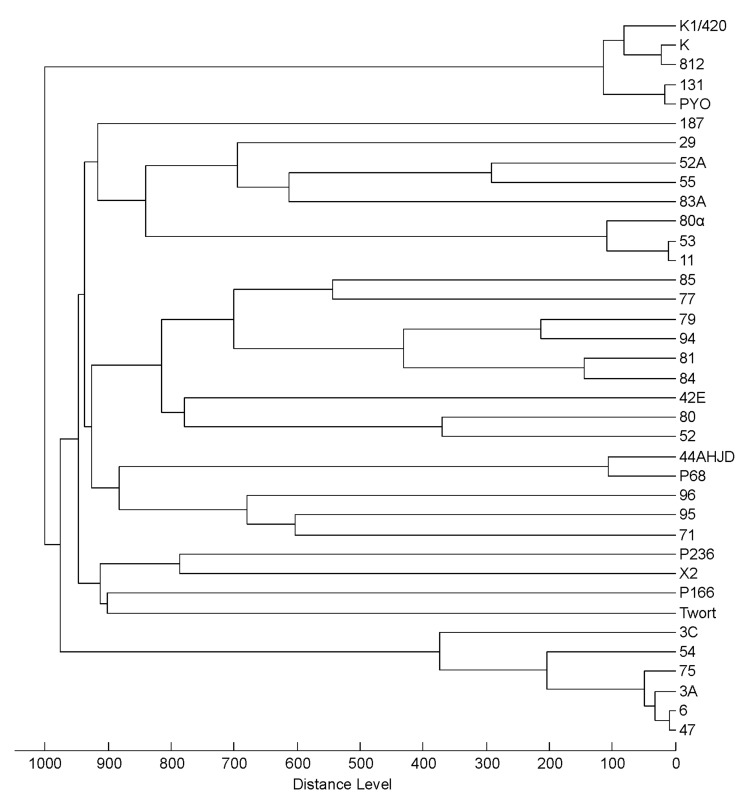
Cluster analysis of mass spectra of all 37 phage strains involved in the study.

Under the sample preparation arrangement proposed, 37 phages were purified and analysed. Mass spectra of *Myoviridae* and *Siphoviridae* were found to be richer than the mass spectra of *Podoviridae*. This could be explained by the fact that *Myoviridae* and *Siphoviridae* include more complex phages with a higher number of structural proteins of molecular weights detectable by MALDI-TOF MS. The dendrogram based on cluster analysis of the mass spectral signals (Figure 5) revealed four significant groups. The first one corresponded to phages of the *Kayvirus* genus (K, K1/420, 812, 131 and PYO). This grouping was supported by visual inspection of the mass spectra where all phage strains shared distinctive peaks at *m/z* = 10,204, 11,533, 15,796, 17,745, 19,112 and 23,067. This mass spectral profile allows unambiguous identification of kayviruses that are frequently used in phage therapy.

Six phages of the second well separated cluster (3A, 3C, 6, 47, 54 and 75) belonged to the *Triavirus* genus and shared several common signals in their MALDI-MS profiles (*m/z* = 7936, 11,645, 12,598, 15,871, 23,295, 25,208 and 30,373). The third well-defined group consisted of transducing phages from the genus *Phietavirus* where phages 11, 53 and 80α are closely related at the genomic level. Mass spectra of other phages from this branch did not show major similarities except phages 29 and 55, that shared the peak at *m/z* = 20,786, corresponding to the major tail protein. The last group consisted of the only two podoviruses P68 and 44AHJD present in this analysis. They shared peaks at *m/z*= 6916, 15,112 and 46,769. The profiles of other phages were not clustered into groups corresponding to their genomic relatedness. MALDI-TOF mass spectra of *Myoviridae* phage Twort (*Twortvirus* genus) and *Siphoviridae* phages 187 and B166 (supposed phietaviruses) showed no similarities with MALDI-TOF mass spectra of any other phages included in the clustered analysis and their genomes and proteomes also differed [29,44].

On the basis of *m/z* values, peaks from mass spectra were assigned to corresponding phage virion proteins annotated in databases (Table 3). The most frequently detected proteins were the major tail protein, Ig-like domain and major capsid protein. Head and tail connecting protein, baseplate protein and various hypothetical proteins were also assigned. In phage K1/420, representing *Kayvirus*, the identity of proteins was confirmed by MALDI-MS/MS analysis of peptides obtained by tryptic digestion of phage K1/420. The masses of proteins listed in Table 4 correspond to those observed in the MALDI-MS profiles.

### 3.4. Practicability of the Method

MALDI-TOF MS is known for its high sensitivity; the detection limit for peptides is reaching, in some cases, attomolar levels. To estimate the detection limit for phages, a series of 10-fold dilutions of CsCl purified phage K1/420 with an initial titre 1 × 10^9^ PFU/mL were prepared. Signals enabling phage identification were detected only in the 10-fold diluted sample. The 100-fold diluted sample yielded only one signal, corresponding to one of the dominant signals of the undiluted phage sample. Further dilution resulted in the absence of any signals (Figure S2). The detection limit of the method is therefore 1 × 10^7^ PFU/mL but practically, 1 × 10^8^ PFU/mL are needed for reliable identification.

To examine the applicability of the method, mass spectra were obtained from pellets formed from 3 mL volumes of lysates of five phages from the initial method evaluation set. Most significantly, phage titre had an impact on mass spectral quality; while no significant decrease in the number of signals compared to the standard procedure was observed for phage strains K1/420 and 3A (initial titre was 7 × 10^9^ and 1 × 10^10^ PFU/mL), the number of signals decreased to approximately 50% for phages 71 and 77 (initial titre was 1 × 10^9^ PFU/mL for both phages) and to less than 30% in the case of phage P68 with an initial titre of 1 × 10^8^ PFU/mL.

To examine the possibility of identifying phages in commercial phage preparations, *Staphylococcus* bacteriophage produced by Microgen, Stafal^®^, Duofag and three individual phages designated SAU1, SAU2 and PAE1 that are components of Duofag, were pelleted from a 20 mL volume and analysed by MALDI-TOF MS. The peak lists obtained were compared to a custom database containing 37 bacteriophage profiles (generated in Section 3.3) using a data treatment approach common to routine bacterial identification using Biotyper software. Similarities in the experimental mass spectra to the individual database entries were expressed by log(scores) that indicate the confidence of identification. For bacterial identification, the log(score) thresholds of 1.700 and 2.000 indicate species identification at lower and higher confidence levels, respectively. From the six phage preparations tested in nine replicates, four samples were assigned to database entries of strains of the *Kayvirus* genus with high confidence (the log(scores) from nine analyses were the following: SAU1: 2.06 ± 0.06, SAU2 2.02 ± 0.05, Stafal^®^: 2.22 ± 0.03 and Duofag: 2.01 ± 0.07) and the remaining sample from the Microgen preparation to the same database entries at a lower confidence level (log(score) = 1.87 ± 0.08). Mass spectra of phage PAE1 did not match any of the database entries (log(score) = 1.07 ± 0.11), which is a true negative result as this phage infects *P. aeruginosa* and differs from staphylococcal phages in our database. It should also be noted that due to the increased number of peaks in the low-mass range, most probably related to a higher complexity of the sample matrix, the identification was based only on signals at *m/z* > 3.5 kDa. Importantly, the score difference between the identification hits of kayviruses and the remaining hits for all samples was always greater than 0.3, which assured no ambiguity in the identification outputs.

## 4. Discussion

Although phage research started over 100 years ago and their therapeutic applications have been studied from the beginning [45], necessary safety requirements for phage preparations are still being discussed [2,46]. Different phage preparations are currently available on the market or under specific experimental treatment programs in Georgia, Poland, Russia, Slovakia and several clinical trials related to phage therapy have been started in the Western World. The production of bacteriophage preparations should comply with recently established requirements including phage identification as a component of quality control [2]. MALDI-TOF MS is among the recommended methods for phage identification in master and working seed lots [46].

Bacteriophage profiling by MALDI-TOF MS described in the literature has mainly been employed in bacterial identification based on phage amplification and detection of specific protein signals. Bacteriophage amplification was also found to be feasible for the identification of components in a bacterial mixture, which is difficult in direct bacterial profiling by MALDI-TOF MS. This was demonstrated by Rees and Voorhees [24] who were able to determine both components of an *E. coli*—*Salmonella* ssp. mixture by detection of proteins from phages MS2 and MPSS-1 inoculated with the bacteria. Cox et al. [47] employed modelling of *Y. pestis* phage φA1122 and *E. coli* MS2 phage amplification to predict optimum growth conditions and thus to simplify the analysis workflow, which normally includes monitoring phage growth by repeated analyses at certain times. To avoid false positive identifications, undetectably low phage titres must be introduced. Therefore, Pierce et al. [48] proposed the use of isotopically labelled 15N *Siphoviridae* bacteriophage 53, whereas the presence of *S. aureus* was based on detection of an unlabelled form of phage 53 capsid protein.

Over the past decade, several variants of the method based on proteomics approaches have been described: MALDI-TOF MS analysis after microwave-assisted acid digestion of MS2 phages isolated by centrifugation and ultrafiltration represents a simple and rapid method [49]. In combination with subsequent digestion by trypsin, MALDI-TOF MS was used for detection of the amplified staphylococcal phage K, in the presence of an antibiotic, where the phage protein tryptic peptides were detected only for antibiotic resistant bacterial strains [50]. Instead of MALDI, electrospray (ESI) has also been employed. After centrifugation, ultrafiltration and acidification, the diluted solution of MS2 protein extract yielded coat protein signals after direct injection to ESI-MS. The identity of the proteins was verified by ESI-MS/MS (Top-down) by Cargile et al. [51] and Wick et al. [52]. An even more sophisticated approach is represented by LC-ESI-MS/MS of phage proteins digested by trypsin [53].

We have shown that pelleting of phages is time- and cost-effective and this technique can be used for sample preparation for MALDI-TOF MS even from a volume of 3 mL of phage lysate containing a sufficiently high titre (approximately 10^9^ PFU/mL). The risk of obtaining limited analytical output when dealing with small sample volumes at lower titres should always be considered, especially when CsCl purification is not involved. The mass spectral quality should be visually monitored to avoid possible ambiguities arising from limited information gained from lower numbers of protein components detected. Similar detection limits were shown by Rees and Barr [50]. Although pelleted phages lack the purity of phages isolated by CsCl density gradient centrifugation, the quality of the MALDI-TOF mass spectra acquired from pellets is comparable to those acquired from CsCl purified phages. At the same time, almost no differences between phages propagated in different types of media or on different propagation strains were found even when prophages were present in the genome of a propagation strain. Prophages are induced spontaneously under various stress conditions [54,55,56]. In our previous work [57] we showed that it was possible to detect spontaneously induced phages by PCR as they can contaminate the lysates of phages propagated on lysogenic strains. The frequency of spontaneous phage induction from lysogenic bacterial strains, as demonstrated by the appearance of infecting phage particles, has been shown to be low—around 10^−8^ to 10^−5^ PFU per bacterial cell [58]. Under experimental conditions, the cell count in cultivation reaches a maximum of 10^8^ cells per mL, therefore 10^3^ induced phages per mL may be present. According to the experimentally set phage detection limit of MALDI-TOF MS (10^7^ PFU/mL), induced phages cannot be detected, particularly in the presence of an excess of propagated phages. Due to ionization suppression effects, MALDI-TOF MS profiling analysis principally determinates only the majority component of the sample. Without employing other separation steps, the method cannot be used for detection of minority components, such as traces of unwanted contaminants of commercial preparations.

The results of cluster analysis of MALDI-TOF mass spectra obtained from 37 phages including 29 *Siphoviridae*, 6 *Myoviridae* and 2 *Podoviridae* did not always correlate with their genome-based taxonomic status. Therefore, the method is not a suitable tool for classification of unknown phage strains to established phage genera. However, due to the satisfactory degree of repeatability of the mass spectra acquired from phages propagated under different conditions, the method can be used for direct phage strain identification or classification to a group of closely related ones. The possibility of reliable phage strain identification will always rely on the uniqueness of signals observed. Strain typing would be practically possible only after comprehensive examination of a wide of range of strains to affirm the specificity of the signals. It is evident from cluster analysis involving three independent cultivations of selected phages (Figure 4), that some strains would not be distinguishable, as was demonstrated on 3A-related phages from *Triavirus* genus. This is due to the fact that the variability of their mass spectra induced by the analytical method itself was greater than differences between mass spectra among different phages within a particular cluster.

Cluster analysis based on MALDI-TOF mass spectra showed that phages from the *Kayvirus* genus were clustered separately from other phages. Distinguishing kayviruses from other phages is important as these phages have been successfully used in the treatment of staphylococcal infections in humans and animals [19,30,59,60,61,62,63]. For most of the tested phages, the MALDI-TOF MS output was strain specific but kayviruses share 99% amino acid identity in most structural virion proteins, therefore they can be considered as variants of one phage strain, where individual mutant variants differ in genes encoding non-structural proteins. It is thus logical that on the basis of their MALDI-TOF MS protein profiles, these strains remain indistinguishable. MALDI-TOF mass spectra with peaks at *m/z* values typical for *Kayvirus* genus were obtained also from pelleted phage preparations. These signals then permitted the identification of strains using a workflow that is familiar to users of MALDI-MS systems in microbial diagnostics. Interpretation of the scoring outputs that was adopted from a routine setup used in bacterial identification represents a field for specific evaluation and probably, different score thresholds should be applied for phage analysis. *Kayvirus* characteristic peaks were found even in the Duofag phage cocktail that consists of two staphylococcal phages and one *P. aeruginosa* phage.

Our findings suggest that MALDI-TOF MS could be used not only for identification of laboratory cultured phages but also in the verification of phages in ready-to-use preparations. The identification success rate is dependent mostly on phage titres, while the cultivation conditions and sample purity do not play a key role. The reliability of identification of individual strains should be evaluated in comparison with other closely related strains.

## Figures and Tables

**Table 1:** List of bacteriophages and staphylococcal strains used in this work.

Family	Serogroup/Genus	Phage ^1^	Propagation Strains (*S. aureus*)
*Siphoviridae*	A/*Triavirus*	3A *	PS 3A, CCM 4890
		3C *	PS 3C
		6 *	PS 6
		42E *	PS 42E
		47 *	RN4220, PS 47, PS 47 [53^+^], PS 47 [77^+^]
		54 *	PS 54
		75 *	PS 75
		79 *	PS 52A
		81 *	PS 81
		94 *	PS 94
	B/*Phietavirus*	11	CCM 4890
		29 *	RN4220
		52 *	PS 52
		52A *	PS 52A, RN4220
		53 *	CCM 4890
		55 *	RN4220
		71 *	PS 71, CCM 4890
		80 *	PS 80
		80α	RN4220
		83A *	PS 83A
		85 *	PS 85, RN4220
		95 *	PS 95
		96 *	PS 96, RN4220
		B166	CCM 4890
		B236	CCM 4890
		X2	CCM 4890
	L/*Phietavirus*	187	PS 187
	F/*Biseptimavirus*	77 *	PS 77, CCM 4890
		84 *	PS 84
*Myoviridae*	D/*Kayvirus*	131	CCM 8428
		812	CCM 4028
		K	RN4220
		K1/420	CCM 8428
		PYO	CCM 8428
	D/*Twortvirus*	Twort	HER 1048 ^2^
*Podoviridae*	G/*P68virus*	44AHJD	RN4220 Δ*tarM*
		P68	RN4220 Δ*tarM*

^1^ Phages belonging to the International Typing Set for human *Staphylococcus aureus* are marked with asterisk (*). ^2^ *Staphylococcus hyicus*.

**Table 2:** Evaluation of the mass spectral quality in terms of signal reproducibility (peaks detected in 100% or 70–99% of mass spectra acquired for each sample) and signal-to-noise ratio of the most intense peak when using five different isolation methods and two matrix-assisted laser desorption ionization (MALDI) matrices.

Phage	Isolation Method	MALDI Matrix	One Term			Three Terms	
			100% Peaks	70–99% Peaks	Maximal Signal-to-Noise	100% Peaks	70–99% Peaks
3A	CsCl gradient	FerA	20	22	504 ± 141	12	18
	CsCl gradient	HCCA	3	2	107 ± 41	-	-
	FPLC	FerA	4	5	34 ± 11	-	-
	Ultrafiltration	FerA	1	8	16 ± 4	-	-
	Pellet	FerA	23	9	517 ± 219	8	8
	Pellet	HCCA	15	4	44 ± 4	-	
71	CsCl gradient	FerA	8	13	397 ± 307	3	15
	CsCl gradient	HCCA	2	2	5 ± 1	-	-
	FPLC	FerA	5	3	157 ± 72	-	-
	Ultrafiltration	FerA	3	6	17 ± 1	-	-
	Pellet	FerA	11	11	557 ± 108	9	4
	Pellet	HCCA	14	9	10 ± 1	-	-
77	CsCl gradient	FerA	10	21	591 ± 96	6	20
	CsCl gradient	HCCA	11	3	64 ± 11	-	-
	FPLC	FerA	0	0	N/A	-	-
	Ultrafiltration	FerA	6	3	19 ± 7	-	-
	Pellet	FerA	12	9	247 ± 36	8	11
	Pellet	HCCA	9	6	15 ± 7	-	-
K1/420	CsCl gradient	FerA	41	13	398 ± 122	21	12
	CsCl gradient	HCCA	17	3	429 ± 30	-	-
	FPLC	FerA	0	0	N/A	-	-
	Ultrafiltration	FerA	0	0	N/A	-	-
	Pellet	FerA	17	7	125 ± 23	8	7
	Pellet	HCCA	5	5	29 ± 3	-	-
P68	CsCl gradient	FerA	13	14	452 ± 122	7	9
	CsCl gradient	HCCA	4	5	281 ± 18	-	-
	FPLC	FerA	19	11	227 ± 23	-	-
	Ultrafiltration	FerA	5	1	41 ± 15	-	-
	Pellet	FerA	9	9	248 ± 19	5	4
	Pellet	HCCA	4	4	399 ± 166	-	-

Legend: N/A, not applicable; -, not analysed.

**Table 3:** List of proteins identified in MALDI-TOF MS spectra.

Phage	NCBI Genome Accession No.	Protein Function	NCBI Protein Accession no.	Mw Theoretical	Mw Experimental
3A	NC_007053	Major tail protein	YP_239944	23,335	23,336
		Ig-like domain	YP_239945	15,855	15,857
		Unknown	YP_239952	10,372	10,373
42E	NC_007052	Major tail protein	YP_239866	23,295	23,290
		Ig-like domain	YP_239868	15,871	15,873
47	NC_007054	Major tail protein	YP_240012	23,295	23,298
		Ig-like domain	YP_240013	15,871	15,875
11	NC_004615	Major tail protein	NP_803292	21,382	21,376
		Head-tail connector protein	NP_803289	12,660	12,658
80α	NC_009526	Major tail protein	YP_001285367	21,395	21,406
		Unknown	YP_001285362	10,790	10,797
53	NC_007049	Major tail protein	YP_239653	21,395	21,405
		Head-tail connector protein	YP_239650	12,660	12,667
55	NC_007060	Major capsid protein	YP_240459	29,487	29,497
71	NC_007059	Head-tail connector protein	YP_240387	11,759	11,758
		Unknown	YP_240388	12,857	12,857
B166	NC_028859	Major tail protein	AKC04659	20,406	20,413
B236	NC_028915	Major capsid protein	YP_009209168	29,489	29,493
187	NC_007047	Major capsid protein	YP_239493	32,997	32,987
77	NC_005356	Major tail protein	NP_958612	23,730	23,731
		Unknown	NP_958619	10,805	10,801
K1/420	KJ206563	Tail tube protein	AHY26502	15,794	15,796
		Baseplate protein	AHY26518	19,109	19,111
		Putative baseplate component	AHY26523	14,480	14,482
		Ig-like protein	AHY26552	23,069	23,070
		Tail morphogenetic protein	AHY26553	17,718	17,718
		Major tail protein	AHY26554	7818	7819
812	KJ206559	Tail tube protein	*	15,794	15,800
		Baseplate protein	AHY25649	19,109	19,114
		Putative baseplate component	AHY25654	14,480	14,479
		Ig-like protein	AHY25683	23,069	23,073
		Tail morphogenetic protein	*	17,718	17,719
		Major tail protein	AHY25685	7818	7817
131	RAST annotations were used	Tail tube protein	*	15,794	15,792
		Baseplate protein	*	19,109	19,109
		Putative baseplate component	*	14,480	14,484
		Ig-like protein	*	23,069	23,069
		Tail morphogenetic protein	*	17,718	17,719
K	NC_005880.2	Tail tube protein	YP_009041323	15,794	15,802
		Baseplate protein	YP_009041339	19,109	19,104
		Putative baseplate component	YP_009041343	14,480	14,488
		Ig-like protein	YP_009041372	23,069	23,071
		Tail morphogenetic protein	YP_009041373	17,718	17,711
Twort	NC_007021	Structural protein	YP_238577	18,759	18,763
		Unknown	YP_238687	3923.5	3920
		Unknown	YP_238618	7638	7639
P68	NC_004679	Major capsid protein	NP_817336	46,769	46,764
		Unknown	NP_817338	15,112	15,104
		Unknown	NP_817337	6916	6915
44AHJD	NC_004678	Major capsid protein	NP_817314	46,769	46,770
		Unknown	NP_817316	15,112	15,110
		Unknown	NP_817315	6916	6917

* Newly sequenced phage genomes annotated using RAST.

**Table 4:** List of proteins identified by MALDI-MS/MS after tryptic digestion of phage K1/420.

NCBI Accession No.	Protein Name	Predicted Protein Function	Mw	No. of Matched Peptides	Mascot Score	Sequence Coverage
YP_240967	ORF189	Major tail protein	7818	8	1276	96%
YP_240933	ORF117	Unknown	14,480	5	291	51%
YP_007112937	F867_gp192	Ig-like protein	23,069	3	188	26%

## Supplementary Materials

The following are available online at http://www.mdpi.com/1999-4915/10/4/176/s1, Figure S1: Comparison of MALDI-TOF mass spectra obtained from phage 3A purified by 500 kDa Pellicon XL 50 ultrafiltration cassette before and after 10 min treatment with β-mercaptoethanol in 1:10 *v*/*v* ratio, Figure S2: MALDI-TOF mass spectra obtained from series of 10-fold dilutions of CsCl purified *Kayvirus* K1/420 with an initial titre 1 × 10^9^ PFU/mL.
