# Supplementary material for: Rapid Identification of Intact Staphylococcal Bacteriophages Using Matrix-Assisted Laser Desorption Ionization-Time-of-Flight Mass Spectrometry
Source: Viruses. 2018 Apr 4;10(4):176. doi: 10.3390/v10040176 (PMC5923470; doi:10.3390/v10040176)
Supplement: Supplementary file 1 [file viruses-10-00176-s001.pdf]

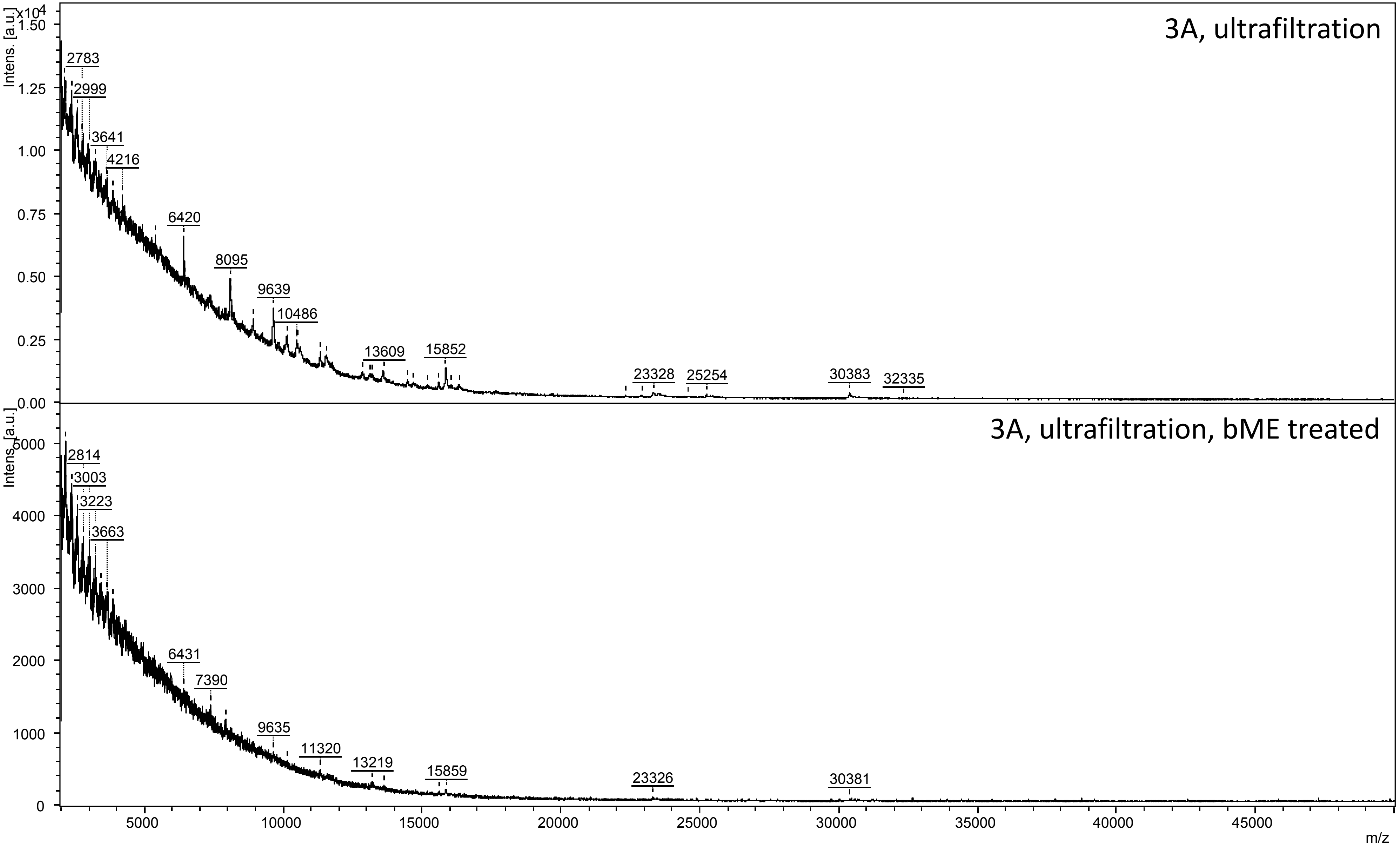

Figure S1. Comparison of MALDI-TOF mass spectra obtained from phage 3A purified by 500 kDa Pellicon XL 50 ultrafiltration cassette before and after 10 min treatment with β-mercaptoethanol (bME) in 1:10 v/v ratio.

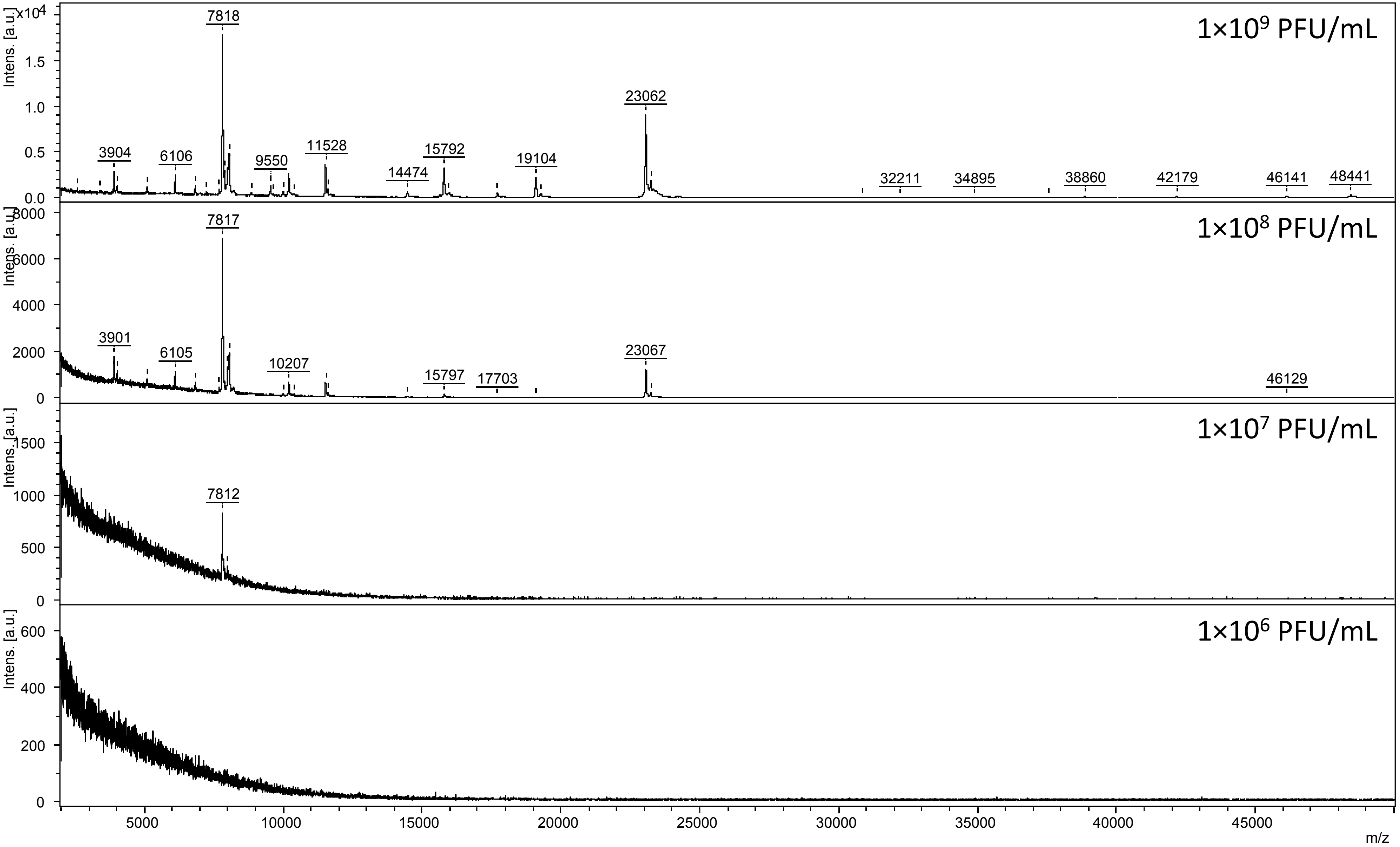

Figure S2. MALDI-TOF mass spectra obtained from series of 10-fold dilutions of CsCl purified *Kayvirus* K1/420 with an initial titer 1×10[9] PFU/mL.
